# Ethnobotanical survey on herbal remedies for the management of type 2 diabetes in the Casablanca-Settat region, Morocco

**DOI:** 10.1186/s12906-024-04468-4

**Published:** 2024-04-15

**Authors:** Maryem Arraji, Nadia Al Wachami, Karima Boumendil, Milouda Chebabe, Latifa Mochhoury, Fatima Zahra Laamiri, Mohamed Barkaoui, Mohamed Chahboune

**Affiliations:** 1 Hassan First University of Settat, Higher Institute of Health Sciences, Laboratory of Sciences and Health Technologies, Settat, 26000 Morocco; 2 Hassan First University of Settat, Institut des Sciences du Sport, Laboratory of Health Sciences and Technologies, Settat, 26000 Morocco

**Keywords:** Phytotherapy, Ethnobotanical survey, Type 2 diabetes mellitus, Medicinal plants, Casablanca-settat, Morocco

## Abstract

**Background:**

Morocco faces a substantial public health challenge due to diabetes mellitus, affecting 12.4% of adults in 2023. The Moroccan population makes extensive use of phytotherapy and traditional medicine to address the difficulties this chronic condition poses. The aim of this study is to document the use of medicinal plants in traditional medicine for managing type 2 diabetes in the provinces of the Casablanca-Settat region.

**Methods:**

The study employed a semi-structured questionnaire for data collection. A study was conducted between August 1st and September 30th, 2023, and 244 individuals diagnosed with diabetes were invited to take part in the research, all of whom used at least one medicinal plant to manage type 2 diabetes, by visiting primary healthcare facilities in Morocco. The analysis included the use of Relative Frequency of Citation (RFC) to scrutinize the data.

**Results:**

A total of 47 plant species belonging to 25 families were documented. Notably, the Apiaceae, Lamiaceae, and Fabaceae families were frequently mentioned in the context of treating type 2 diabetes in Morocco. Prominent among the cited plant species were *Sesamum indicum* L., *Lepidium sativum* L., followed by *Foeniculum vulgare* Mill., and *Rosmarinus officinalis* L. Seeds emerged as the plant part most commonly mentioned, with infusion being the prevailing preparation method and oral consumption being the most frequently depicted method of administration.

**Conclusion:**

This research underscores the practicality of incorporating traditional medicine into the healthcare framework of the Casablanca-Settat region. The findings not only offer valuable documentation but also have a vital function in safeguarding knowledge regarding the utilization of medicinal plants in this locality. Moreover, they provide opportunities to delve deeper into the phytochemical and pharmacological potential of these plants.

## Background

Diabetes Mellitus (DM) represents a health imbalance marked by metabolic dysfunction, culminating in a continual increase in blood glucose levels. In accordance with the guidelines provided by the World Health Organization (WHO) in 2023, this situation arises due to disturbances in the secretion of insulin, Effectiveness of insulin action, or a combination of both [1]. DM can be generally classified into two categories: type 1, also known as juvenile or insulin-dependent diabetes mellitus, with a hereditary basis requiring insulin treatment; and type 2 (T2DM), also termed adult-onset or diabetes mellitus not requiring insulin, influenced by dietary habits or the use of medications for oral administration aimed at reducing blood glucose levels [2]. DM poses a significant global community health challenge, affecting millions and leading to serious complications such as, for example hypertension, hyperinsulinemia, atherosclerosis, and hyperlipidemia [3, 4]. According to the International Diabetes Federation (IDF) atlas for 2021, the global prevalence of diabetes stands at 537 million people, with three out of four adults living in low- or middle-income countries [5]. In Morocco, in accordance with the WHO in 2023, approximately 12.4% of Moroccan adults are reported to have this chronic condition, and this non-communicable disease is the cause of over 12,000 annual deaths, with an additional association with 32,000 deaths resulting from complications due to elevated blood sugar levels [6]. T2DM has a significant socio-economic impact in Morocco. In terms of health, it leads to an increase in medical expenses, notably due to chronic complications such as neuropathy, retinopathy, renal failure, heart attacks, or strokes [1]. These complications require costly treatments and often result in an inability to work and perform daily tasks, which has direct economic repercussions on the affected individuals, their families, and society as a whole [7]. This situation leads to a deterioration in the patient’s health status, increases the risk of treatment failure and hospital visits, resulting in a financial burden for both the patient and the healthcare system [8]. Several factors contribute to the incidence of T2DM, particularly the consumption of high-fat or sugary foods, lifestyle changes, obesity, imbalanced diets, and sedentary behavior [9, 10]. The administration of T2DM involves the utilization of a variety of antihyperglycemic agents, among which various medicinal plants are utilized. Recently, even in the most industrially advanced nations, traditional healthcare treatments have gained remarkable recognition due to their affordability in contrast to conventional medicinal medications, and the perceived lower occurrence of side impacts by diabetic patients [11, 12]. According to the WHO in 2010, exceeding 80% of the global population, particularly among developing countries, relies on the use of medicinal plants to address their main healthcare needs [13]. Additionally, a minimum of 25% of pharmaceutical medications are derived from plants [14]. The ways in which these plants impact blood glucose levels can vary; some demonstrate effects comparable to pharmaceutical antidiabetic medications like sulfonylureas, hepatic gluconeogenesis inhibitors, or glucosidase inhibitors [15]. Additionally, research indicates that the concurrent utilization of medicinal plant extracts and their constituents can synergistically enhance effectiveness in treating T2DM [16]. Plant-based treatments are traditionally preferred due to various advantages, not only because of reduced cost but also for their accessibility and effectiveness as demonstrated by the experiences of these patients and their families [17–20]. Thus, primary healthcare significantly benefits from remedies derived from local traditional medicine [18, 21]. Following various previous surveys, it emerges that diabetic patients have a keen interest in herbal traditional medicine. Furthermore, these studies emphasize the established efficacy of medicinal plants in the management of this chronic disease [22–25]. Morocco is famous for its abundant greenery and exceptional plant diversity, boasting a total of 5,200 species and classifications of vascular plants, of which 900 are native [26]. Morocco’s flora encompasses around 600 species of medicinal plants [27]. These plants have a longstanding history of medicinal use in Morocco and remain integral to healthcare practices. Moroccan diabetic patients often turn to traditional medicine for treatment [18, 23, 28, 29]. In the Casablanca-Settat region, ethnobotanical studies are quite limited. In 2012, Nabila TAHRI and her team conducted an ethnobotanical study into the utilization of medicinal plants within the general population of Settat province, the study inventoried 90 species belonging to 44 different plant families. Most of these species are used primarily in the care of the digestive and respiratory systems, with 11.5% of these species being used for the treatment of metabolic disorders, including type 2 diabetes [30]. Another study conducted in several localities in the Casablanca-Settat region by Essaih et al. (2021), also involving the general population, revealed that the species used in the treatment of diabetes accounted for 7.31% of all species listed [31]. Our current study took place in primary healthcare facilities in the Casablanca-Settat region, Morocco. This initiative was motivated by the absence, to our knowledge, of previous surveys regarding the utilization of medicinal plants among individuals diagnosed with T2DM. Therefore, the importance of this study resides in its capacity to address this deficiency by pinpointing the species of medicinal plants employed in traditional medicine for treating T2DM among patients seeking primary healthcare services in the specified region of Morocco.

## Methods

### Study area

**Fig. 1:**
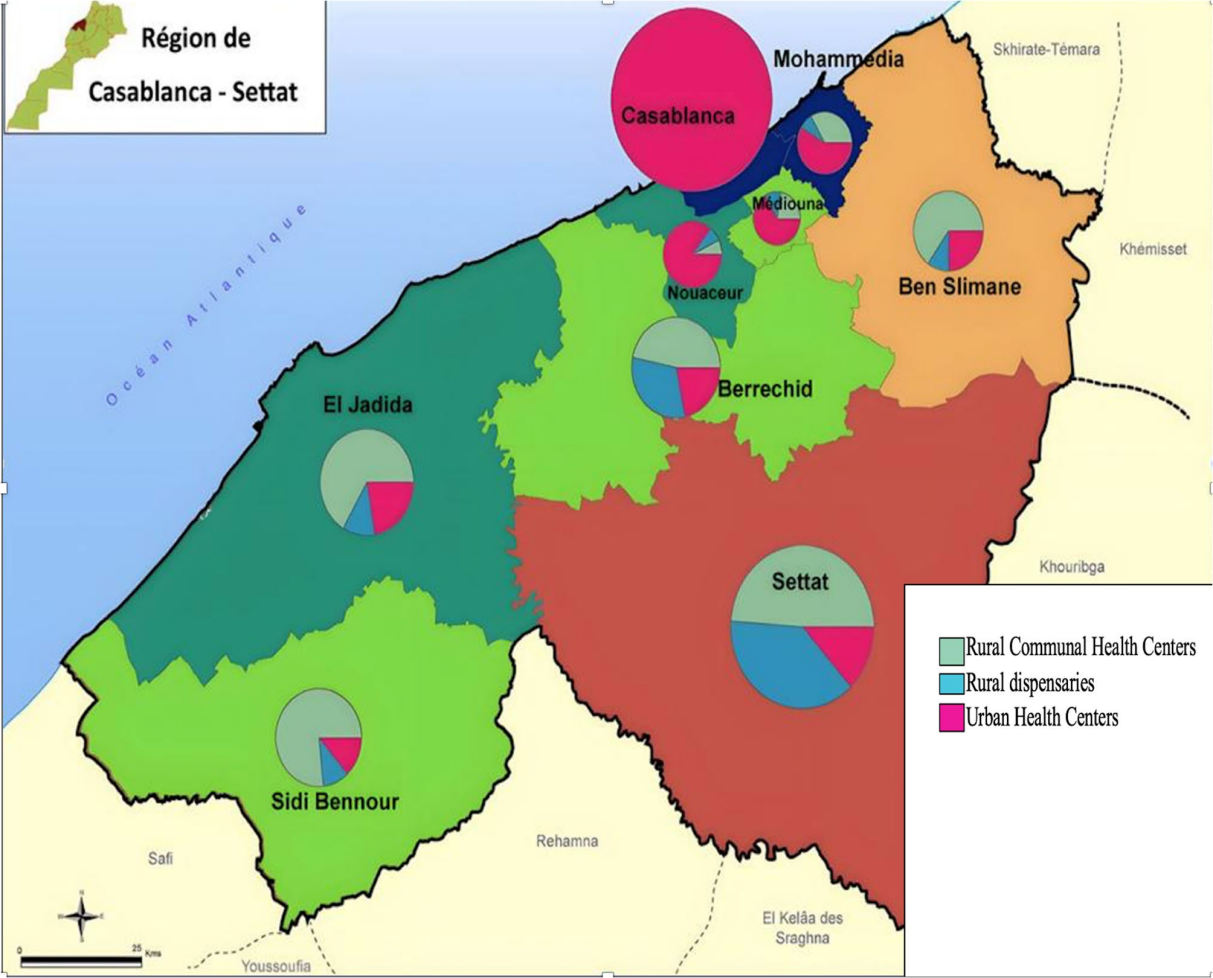
The study area includes primary healthcare facilities in the region, as defined by the Ministry of Health and Social Protection in 2013

Our study took place in the Casablanca-Settat region (Fig. 1), encompassing an area of approximately 20,394 square kilometers. This region is situated between the Atlantic Ocean to the northwest, the southern Marrakech-Safi region and the eastern Beni Mellal-Khenifra region. With a population of 6,861,739 inhabitants, it constitutes 20.3% of the national population. The climate in this area is influenced by the Atlantic Ocean, with its intensity diminishing as one moves inland. Generally, the region exhibits a semi-arid to arid climate, featuring a gradual decline in precipitation from north to south, as reported by the Ministry of Agriculture, Maritime Fisheries, Rural Development, and Water and Forests in 2018. The Casablanca-Settat region is notable for its abundant plant biodiversity, growing a variety of medicinal and aromatic plants traditionally used in herbal medicine by knowledgeable herbalists, informed community members, and practitioners of traditional healing methods.

### Ethnobotanical survey

The ethnobotanical study took place between August 1st and September 30th,2023. Data were collected through a questionnaire, primarily in Moroccan Arabic, which represents the local language of the study region. We collected the names of medicinal plants utilized for treating T2DM, specifically in Moroccan Arabic, as mentioned by individuals with T2DM in our study. This study involved a total of 244 participants attending healthcare centers at the primary level in Morocco, where both programs for both treating and preventing care are offered for individuals experiencing chronic conditions. The participants in the study were individuals aged 18 years and above, having prediabetes and/or previously diagnosed diabetes, with or without additional health conditions, and receiving antidiabetic treatment for a minimum of one month prior to their participation in the study. Additionally, they were using at least one medicinal plant to treat their diabetes. Exclusion criteria included patients scheduled for surgery, pregnant women, or those experiencing acute health problems requiring immediate medical attention.

The objective of this study was to collect information on plants employed in the treatment of T2DM, including their vernacular names, preparation methods, plant parts used, participants’ sociodemographic characteristics, and information pertaining to their diabetic condition.

The recognition and identification of therapeutic plants obtained from the involved patients after the ethnobotanical survey were initially conducted by a botanist. Then, the confirmation and updating of the scientific names are done using the national flora “Flore du Maroc,” [32–34] along with the link for the update, available at http://www.ipni.org/.

### Data analysis

The responses gathered from the survey were documented and organized in a Microsoft Excel database. Subsequently, these data were scrutinized to ascertain the proportions of various variables. The examination involved both comparative and descriptive analyses. Additionally, a specific evaluation was performed using the ethnobotanical index known as the Relative Frequency of Citation (RFC) to gauge the regional significance for each individual species.

RFC is calculated based on the local therapeutic importance of each plant species. It is obtained by dividing the number of participants who mentioned the use of a particular plant species (Fc) by the total number of participants (N), according to the following formula [35]: RFC = Fc/N.

## Results and discussion

### Sociodemographic and diabetes-related information

The main objective of this research was to ascertain the healing plants employed for the management of T2DM in the Casablanca-Settat region. Table 1 presents sociodemographic characteristics and data related to T2DM. Our findings underscore the region’s deep connection to traditional medicine, as evidenced by the participants’ extensive familiarity with the botanical legacy and cultural insights into medicinal plants. A cohort of 244 individuals, averaging 58.56 ± 13.405 years in age and ranging from 29 to 95 years, were surveyed in the reaserch locale. The majority (73.35%) fell within the 50 years and older age group, indicating a more profound knowledge of plants compared to younger individuals, which aligns with similar observations in prior studies [36–38]. However, this does not discount the potential understanding of medicinal herbs among other age groups. Slightly over half of the participants resided in rural areas (56.1%), and 59% had no formal education, consistent with findings from other studies highlighting a preference for traditional medicine among the illiterate [39–43]. Among the participants, 126 were men (51.6%) and 118 were women (48.4%), indicating a slight male predominance, which contrasts with some earlier studies emphasizing an elevated prevalence of medicinal plants use among women [43, 44]. Moreover, 61.88% of the participants were unemployed, and 87.29% had health insurance. Concerning their health, 69.3% had a disease duration of 1 to 10 years, 53.7% practiced self-monitoring of blood glucose levels, 65.57% had an apparent comorbidity, 52% were informed about their diabetes, and 78.69% did not engage in any physical activity. Regarding herbal medicine knowledge, the majority of participants (74.59%) acquired it inside their own family, while others obtained it from sources outside their family sphere.

**Table 1:** Sociodemographic characteristics and diabetes-related data

	N	Percentage(%)
**Sociodemographic data**		
**Gender**		
Female	118	48.4
Male	126	51.6
**Age**		
25–49	65	26.65
50–70	124	50.81
71–95	55	22.54
**Place of residence**		
Urban	107	43.9
Rural	137	56.1
**Marital status**		
Married	28	11.5
Widowed	168	68.9
Divorced/Single	48	19.7
**Educational level**		
Illiterate	144	59
Primary/Secondary School	78	32
High School/University	22	9
**Occupational status**		
Not employed	151	61.88
Retired	64	26.22
Employed	29	11.88
**Health insurance**		
Yes	213	87.29
No	31	12.71
**Diabetes-related data**		
**Duration of the disease**		
Less than a year	14	5.7
1 to 10 years	169	69.3
11 to 15 years	23	9.4
16 years and above	38	15,5
**Practice of self-monitoring of diabetes**		
Yes	131	53.7
No	113	46.3
**Presence of comorbidities**		
Yes	160	65.57
No	84	34.63
**Engagement in physical activity**		
Yes	52	21.31
No	192	78.69
**Patient’s awareness of their condition**		
Yes	127	52
No	117	48
**Source of knowledge on herbal medicine**		
Family heritage	182	74.59
Divine revelation	32	13.11
Traditional initiation	21	8.6
Other	9	3.68

### Plants with antidiabetic properties

According to the results obtained from the questionnaires, the utilization of 47 plant species by the residents of the region for the management of T2DM has been highlighted. These plants, used for medicinal purposes against this chronic disease have been classified into 25 families.

**Fig. 2:**
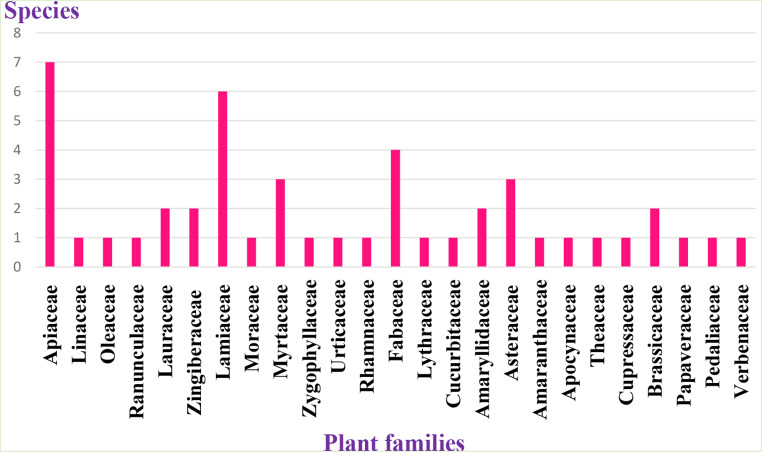
The number of species within each family

Figure 2 shows the number of species in each family, with Apiaceae having the highest number of species with 7 plants, followed by Lamiaceae (6 plants), and Fabaceae (4 plants).

This dominance of Apiaceae has been observed in Togo [45] and Benimellal [39], unlike what has been observed in another region of Morocco [44].

**Fig. 3:**
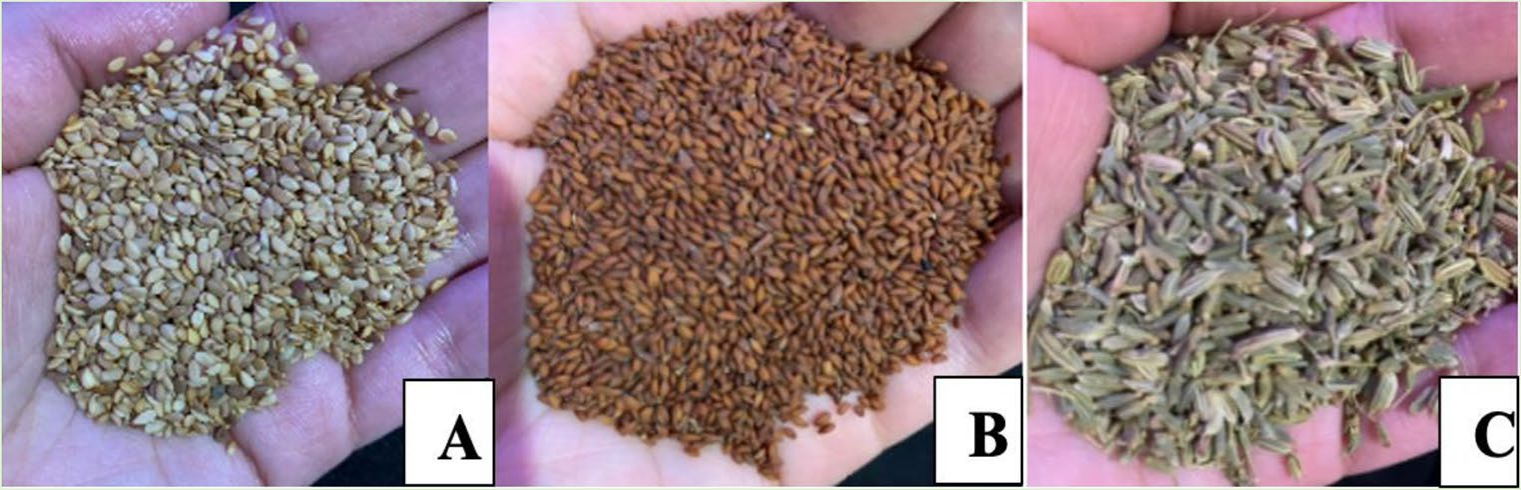
Images of (**A**) *Sesamum indicum* L., (**B**) *Lepidium sativum* L., (**C**) *Foeniculum vulgare* Mill

Table 2 illustrates the plants used in the Casablanca-Settat regions for managing T2DM and its complications. In our current study, the most frequently used species were *Sesamum indicum* L., with the highest RFC at 9.8%, closely followed by *Lepidium sativum* L., *Foeniculum vulgare* Mill, and *Rosmarinus officinalis* L., having RFC values at 7%, 6.10%, and 5.7%, respectively. The ethnobotanical study carried out in Benimellal in 2019 revealed a prevailing utilization of *Olea europaea*, exhibiting the highest RFC at 24.3%. *Salvia officinalis*, followed by *Allium sativum*, and *Trigonella foenum-graecum* emerged as closely-following contenders, With impressive RFC measurements of 23.0%, 22.5%, and 20.5%, correspondingly. In contrast, Barkaoui and colleagues [44] identified *Allium sativum*, subsequently by *Salvia officinalis*, followed by *Marrubium vulgare*, and *Lavandula dentata* as the greatest commonly referenced vegetation for T2DM treatment, featuring RFC values of 0.29%, 0.31%, 0.29%, and 0.64%, in that order. Additionally, a research initiative in southern Morocco highlighted *Allium cepa, Ajuga iva*, *Carum carvi, Lepidium sativum, Artemisia herba-alba*, *Olea europaea, Nigella sativa, Phoenix dactylifera, Peganum harmala, Zygophyllum gaetulum* and *Rosmarinus officinalis*, as frequently cited plants in traditional medicine for T2DM treatment [22]. The images of the 3 species with the highest RFC are illustrated in Fig. 3.

**Table 2:** Plants utilized in the Casablanca-Settat regions for managing type 2 diabetes mellitus

Families	Plant species	Vernacular name	Plant part used	Preparation form	Mode of administration	RFC (%)	References in literature regarding the medicinal utilization of plants for managing T2DM	
							In Morocco	Out of Morocco
**Amaranthaceae**	*Chenopodium ambrosioides* L.	**Lmkhinza**	L	Mac	Oral	**2.5**	[39, 46–52]	**No Data**
**Amaryllidaceae**	*Allium sativum* L.	**Toma**	Bu	Raw	Oral	**0.4**	[22, 23, 39, 46, 48–53]	[10, 54–58]
	*Allium cepa* L.	**Bessla**	Bu	Raw	Oral	**2.5**	[22–24, 39, 44, 46–51, 53, 59]	**No Data**
**Apiaceae**	*Coriandrum sativum* L.	**Kesbour**	Se L	Inf	Oral	**1.2**	[24, 46, 47, 53, 59]	**No Data**
	*Pimpinella anisum* L.	**Hebbat -helawa**	Se	Inf	Oral	**0.8**	[18, 39, 44, 46, 48, 53]	**No data**
	*Petroselinum crispum* (Mill.) Fuss	**Maadnous**	L St	Dec	Oral	**0.8**	[39, 46, 48, 53]	**No Data**
	*Carum carvi* L.	**karwiya**	Se	Inf	Oral	**0.4**	[21, 22, 24, 39, 44, 48, 53, 60]	[54, 61]
	*Foeniculum vulgare* Mill.	**Nafea**	Se	Dec	Oral	**6.1**	[18, 22–24, 39, 44, 48, 49, 60]	[54]
	*Pastinaca sativa* L.	**Laft-mahfor**	R	Raw	Oral	**3.7**	[39, 44]	**No Data**
	*Ammi visnaga* (L.) Lam	**Bouchnikha**	Fr	Dec	Oral	**0.8**	[22–24, 46, 47, 49–53]	**No Data**
**Apocynaceae**	*Nerium oleander* L.	**Deflla**	L	Fum	Inhalation	**04**	[22–24, 39, 44, 46–49, 51–53, 59, 62]	**No Data**
**Asteraceae**	*Artemisia herba-alba* Asso.	**Echih**	L St	Inf Dec	Oral	**2.9**	[53]	[54, 63, 64]
	*Matricaria chamomilla* L.	**Babounej**	Fl	Inf	Oral	**0.4**	[53, 59]	**No Data**
	*Artemisia absinthium* L.	**Shiba**	L	Dec Inf	Oral	**3.7**	[22–24, 39, 46, 47, 49, 51–53]	**No Data**
**Brassicaceae**	*Raphanus sativus* L.	**Fjel**	R	Raw	Oral	**4.4**	[39, 44, 46, 48, 53]	[65]
	*Lepidium sativum* L.	**Habberchad**	Se	Inf	Oral	**7.0**	[22, 39, 46, 48, 49, 53]	[54]
**Cucurbitaceae**	*Cucumis sativus* L.	**Khyar**	Fr	Raw	Oral	**0.4**	[39, 44, 46, 48, 50, 53]	[65]
**Cupressaceae**	*Tetraclinis articulata* (Vahl) Mast	**Araar**	L	Mac	Oral	**0.4**	[22, 23, 39, 46, 50–53, 59]	**No Data**
**Fabaceae**	*Medicago sativa* L.	**Fessa**	Se	Pow	Oral	**0.4**	[39, 46, 48, 50, 53]	**No Data**
	*Trigonella foenum-graecum* L.	**Halba**	Se	Mac	Oral	**3.3**	[22–24, 39, 44, 46–53, 59, 62]	[54–57, 63, 65–67]
	*Glycyrrhiza glabra* L.	**Arksouss**	Ba	Inf Raw	Oral	**2.9**	[68]	**No Data**
	*Ceratonia siliqua* L.	**Kherroub**	Fr	Raw	Oral	**0.4**	[39, 44, 46, 48, 53]	**No Data**
**Lamiaceae**	*Marrubium vulgare* L.	**Mriwt/Mrriwa**	L St	Inf	Oral	**0.8**	[22, 23, 39, 44, 46–52, 60]	[54, 63]
	*Salvia officinalis* L.	**Salmiya**	L	Inf	Oral	**0.8**	[23, 39, 44, 46–53, 59, 62]	[54]
	*Origanum compactum* Benth.	**Zaâter**	L St	Inf Mac	Oral	**2.9**	[51]	**No Data**
	*Rosmarinus officinalis* L.	**Yazir**	L St	Inf Dec Mac	Oral	**5.7**	[22, 23, 44, 48–53, 59]	**No Data**
	*Ajuga iva* (L.) Schreb.	**Chndgoura**	L	Pow	Oral	**1.2**	[22, 23, 39, 44, 46, 48–53, 59, 62]	[54, 63, 66, 69]
	*Calamintha officinalis* Moench	**Minta**	L St	Dec Inf	Oral	**1.6**	[21, 39, 44, 47–51, 53, 59–61]	**No Data**
**Lauraceae**	*Cinnamomum verum* J. Presl	**kerfa**	Ba	Mac Dec	Oral	**3.3**	[22, 49, 50, 53, 59]	**No Data**
	*Laurus nobilis* L.	**Awrak Rand**	L	Inf	Oral	**0.4**	[46, 53]	**No Data**
**Linaceae**	*Linum usitatissimum* L.	**Zerriat-lkattan**	Se	Pow	Oral	**3.3**	[22, 44, 48, 50, 53, 59]	**No Data**
**Lythraceae**	*Punica granatum* L.	**Romman**	Ba	Dec	Oral	**0.4**	[22, 24, 44, 48–50, 53, 59]	[54, 57, 63, 70]
**Moraceae**	*Ficus carica* L.	**Chriha**	L	Inf	Oral	**0.4**	[23, 24, 39, 44, 50, 52, 53, 59]	[65]
**Myrtaceae**	*Eucalyptus camaldulensis* Dehnh.	**Kaliptouss**	L	Dec	Oral	**4.1**	[48]	**No Data**
	*Syzygium aromaticum* (L.)	**Qrenfel**	Se	Pow	Oral	**0.4**	[23, 39, 44, 48, 49, 51, 53]	**No Data**
	*Myrtus communis* L.	**Rihane**	L	Dec	Oral	**2.5**	[22, 24, 39, 50–53]	[71]
**Oleaceae**	*Olea europea* L. subsp. *europaea* var. *sylvestris* (Mill) Lehr,	**Jebbouj**	L	Dec	Oral	**0.4**	**No Data**	**No Data**
**Papaveraceae**	*Papaver rhoeas* L.	**Belàman**	Se	Pow	Oral	**5.3**	[39, 44, 62]	[63]
**Pedaliaceae**	*Sesamum indicum* L.	**Jenjlane**	Se	Inf Pow	Oral	**9.8**	[22–24, 39, 48, 51]	**No Data**
**Ranunculaceae**	*Nigella sativa* L.	**channouj/habba -sawdae**	Se	Pow	Oral	**0.4**	[22–24, 39, 44, 46–48, 50–53, 59, 62]	[54, 65, 72]
**Rhamnaceae**	*Ziziphus lotus* (L.) Lam.	**Ssedra**	L	Dec Pow	Oral	**5.3**	[23, 24, 39, 44, 48, 49, 52, 53, 59]	[54, 63]
**Theaceae**	*Camellia sinensis* (L.) Kuntze	**Atay**	L	Inf	Oral	**1.2**	[39, 44, 46, 53]	**No Data**
**Urticaceae**	*Urtica urens* L.	**Hrriga**	L St	Dec	Oral	**0.8**	**No Data**	**No Data**
**Verbenaceae**	*Verbena officinalis* L.	**Lwiza**	L	Dec	Oral	**0.8**	[68]	**No Data**
**Zingiberaceae**	*Curcuma longa* L.	**kharkoum**	Rh	Inf	Oral	**0.4**	[68]	**No Data**
	*Zingiber officinale* Roscoe	**Skinjbir**	Rh	Mac	Oral	**0.4**	[48, 49, 51, 59]	**No Data**
**Zygophyllaceae**	*Peganum harmala* L.	**Harmal**	Se	Mac	Oral	**1.6**	[22–24, 50, 52, 53, 59]	[54, 67]

RFC: relative frequency of citation; L: leaves; Se: Seed; Bu: bulb; St: stem; R: root; Fl: flower; Fr: fruit; Inf: infusion; Pow: powder; Dec: decoction; Rh: Rhizomes; Mac: maceration; Ba: bark; Fum: fumigation.

### Used parts, preparation methods, and modes of administration

**Fig. 4:**
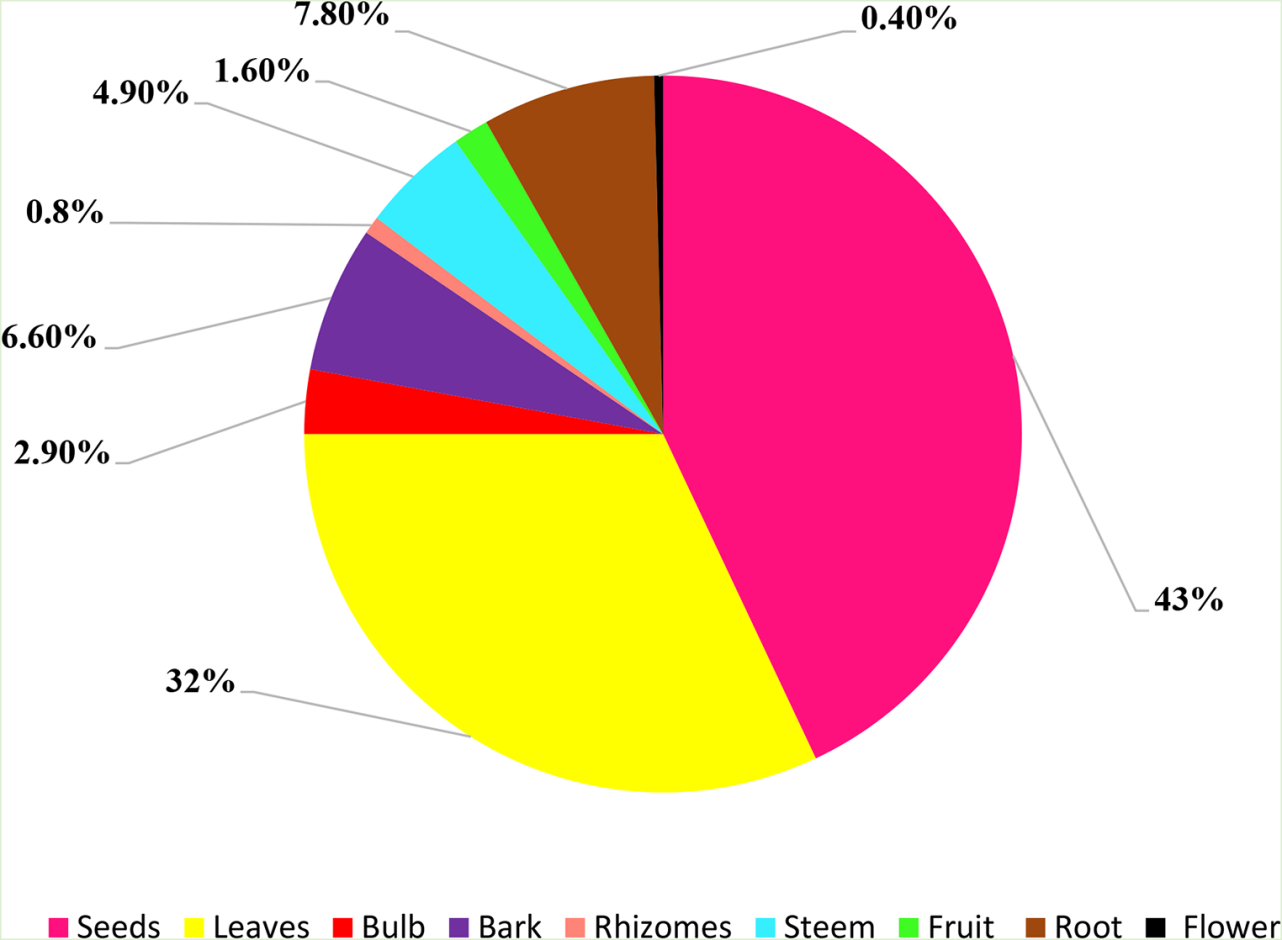
Distribution of plants parts used (%)

Figure 4 presents the percentage of plant parts used in the present study. Seeds emerged as the most commonly utilized plant component (43%) among medicinal plants, with leaves following closely (32%) and roots (7.8%). This contrasts with previous findings in Khénifra [73], Pakistan [65], Kenya [10], and Togo [36], which suggested that leaves are the plant component most frequently employed. The utilization of leaves in treating diseases depends on their availability and therapeutic substance richness, and so, the collection remains easier compared to fruits, roots, or flowers [74].

**Fig. 5:**
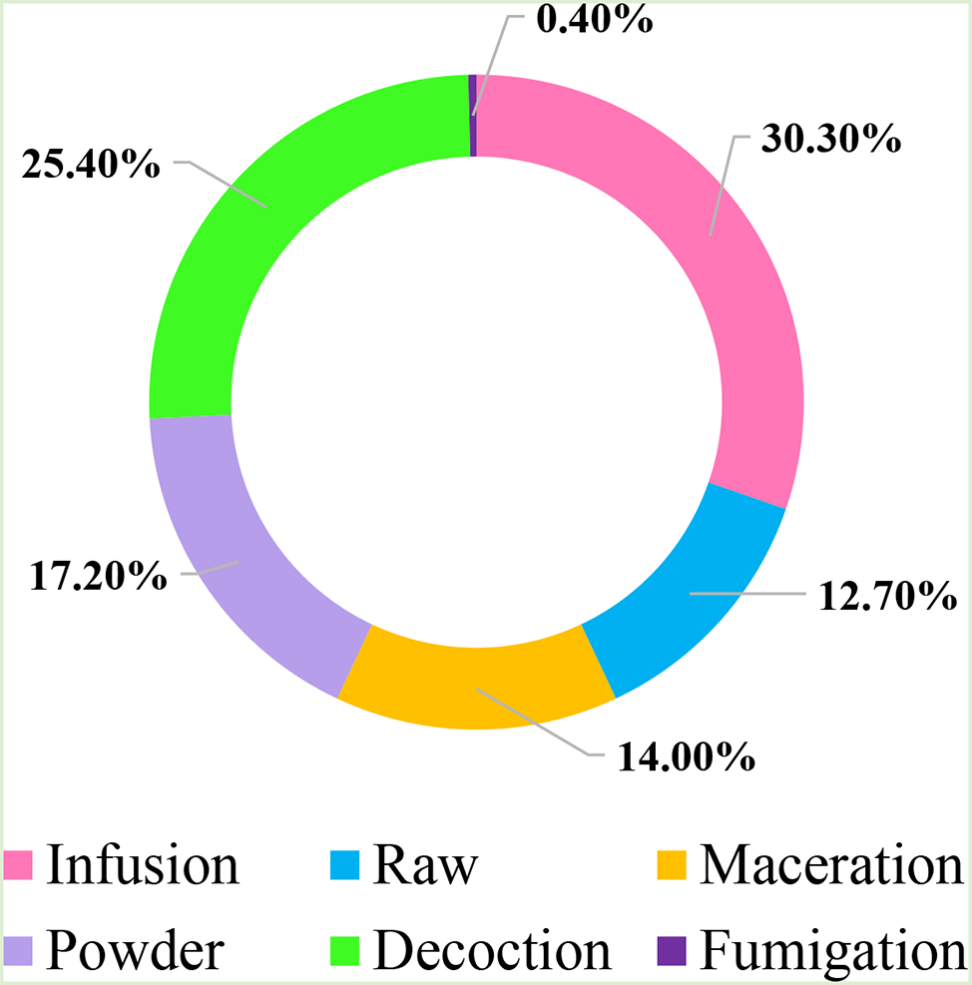
The percentage of different preparation modes

Figure 5 illustrates the percentage of different preparation modes. The preparation techniques for medicinal extracts varied among sociocultural groups. In our context, infusion was the most common preparation method for herbal remedies (30.3%), followed closely by decoction (25.4%) and powder (17.2%). These results contrast with another ethnobotanical study conducted in Algeria [75], indicating that preparations are generally made by decoction, accounting for approximately 89.23% of cases, as observed in previous studies [39, 44].

It was noted that water is frequently used as a solvent in most herbal remedy preparations, explained by its availability and ease of access.

**Fig. 6:**
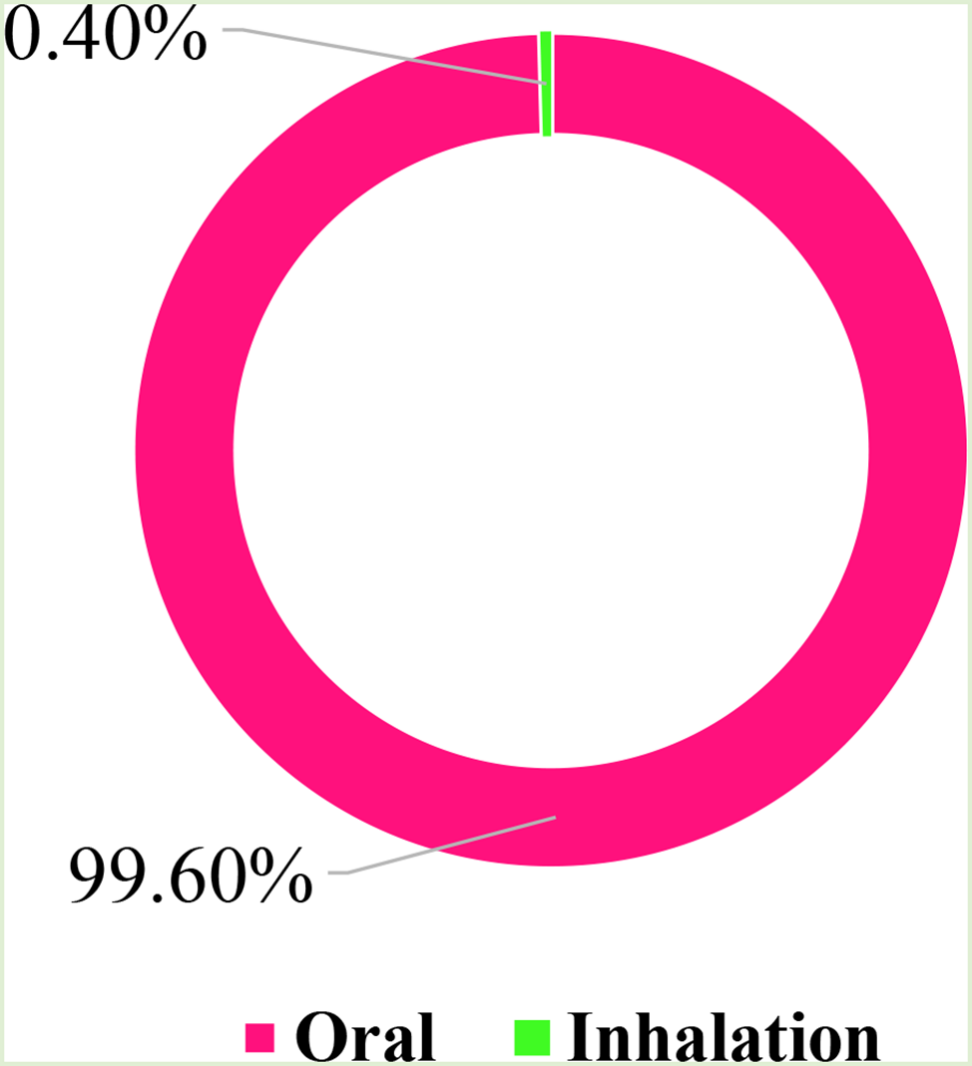
Method of administration

Figure 6 highlights the percentage of administration methods for preparations of these plant species used in the management of T2DM among our target population. The overwhelming majority of these observed treatments were administered orally (99.6%), consistent with results from other studies [70, 76], indicating that these plants are commonly ingested as tea or infusion.

The findings from our study have provided intriguing information about antidiabetic medicinal plants, highlighting their potential for future research in the development of antidiabetic drugs.

## Conclusion

In total, the study revealed 47 floral species originating from 25 families. Apiaceae, Lamiaceae, and Fabaceae families were frequently highlighted for their role in treating T2DM in the research region. Notable among the specified plant species were *Sesamum indicum* L., *Lepidium sativum* L., *Foeniculum vulgare* Mill., and *Rosmarinus officinalis* L. Medicinal knowledge was shared by both genders, with a slight inclination towards men in the utilization of these medicinal plants. The predominant choice for plant parts used in remedies was seeds. Infusion was the most commonly practiced pharmaceutical form by the local population, and oral administration remained their preferred method. This study provided insights into the traditional medicine practices for treating this chronic disease, as conveyed by the population in this region. This ethnopharmacological research emphasized the region’s wealth of diverse plant species utilized in alternative medicine. Indeed, these traditional healing practices ought to be endorsed by specialized services, and their use should be guided by favorable data regarding their efficacy and safety before being employed by patients. However, additional pharmacological, toxicological, and conducting phytochemical investigations becomes imperative to substantiate or challenge their clinical application. It is also desirable to standardize the correct therapeutic doses for these patients in order to prevent dangers associated with poisoning caused by doses that do not conform to regulations. Nevertheless, several limitations were identified during this survey. Notably, some participants chose confidentiality regarding certain plants, considering them as personal secrets, which slightly hindered the smooth transmission of this ancestral knowledge. In this regard, we suggest involving herbalists and specialists to deepen the collection of this information and extend the study to all other regions of Morocco.

## Data Availability

All data generated or analysed during this study are included in this published article.

## Abbreviations

Ba: Bark
Bu: Bulb
Dec: Decoction
DM: Diabetes Mellitus
Fl: Flower
Fr: Fruit
Fum: Fumigation
IDF: International Diabetes Federation
Inf: Infusion
L: Leaves
Mac: Maceration
Pow: Powder
R: Root
RFC: relative frequency of citation
Rh: Rhizomes
Se: Seed
St: Stem
T2DM: Type 2 Diabetes Mellitus
WHO: World Health Organization
